# Antimicrobial, Antioxidant, and Anti-Inflammatory Activities of Essential Oils of Selected Aromatic Plants from Tajikistan

**DOI:** 10.3390/foods4040645

**Published:** 2015-11-02

**Authors:** Farukh Sharopov, Markus Santhosh Braun, Isomiddin Gulmurodov, Davlat Khalifaev, Salomiddin Isupov, Michael Wink

**Affiliations:** 1 Institute of Pharmacy and Molecular Biotechnology, Heidelberg University, Im Neuenheimer Feld 364, Heidelberg 69120, Germany; E-Mails: sharopov@uni-heidelberg.de (F.S.); m.braun@uni-heidelberg.de (M.S.B.); 2 Tajik State Medical University, Rudaki Str. 139, Dushanbe 734025, Tajikistan; E-Mails: gulmurodov@mail.ru (I.G.); nifc@list.ru (D.K.); salomidin@mail.ru (S.I.)

**Keywords:** essential oil, antioxidant activity, aromatic plant, anti-inflammation, antimicrobial activity

## Abstract

Antimicrobial, antioxidant, and anti-inflammatory activities of the essential oils of 18 plant species from Tajikistan (Central Asia) were investigated. The essential oil of *Origanum tyttanthum* showed a strong antibacterial activity with both minimum inhibitory concentration (MIC) and minimum bactericidal concentration (MBC) values of 312.5 µg/mL for *E. coli*, 625 µg/mL (MIC) and 1250 µg/mL (MBC) for MRSA (methicillin-resistant *Staphylococcus aureus*), respectively. The essential oil of *Galagania fragrantissima* was highly active against MRSA at concentrations as low as 39.1 µg/mL and 78.2 µg/mL for MIC and MBC, respectively. *Origanum tyttanthum* essential oil showed the highest antioxidant activity with IC_50_ values of 0.12 mg/mL for ABTS (2,2′-azino-bis-(3-ethylbenzthiazoline-6-sulfonic acid)) and 0.28 mg/mL for DPPH (2,2-diphenyl-1-picrylhydrazyl). *Galagania fragrantissima* and *Origanum tyttanthum* essential oils showed the highest anti-inflammatory activity; IC_50_ values of 5-lipoxygenase (5-LOX) inhibition were 7.34 and 14.78 µg/mL, respectively. In conclusion, essential oils of *Origanum tyttanthum* and *Galagania fragrantissima* exhibit substantial antimicrobial, antioxidant, and anti-inflammatory activities. They are interesting candidates in phytotherapy.

## 1. Introduction

Essential oils consist of mostly volatile and small lipophilic secondary metabolites comprising hydrocarbons (terpenes and sesquiterpenes) and oxygenated compounds (alcohols, aldehydes, ketones, phenols, ethers, esters, lactones, and phenol ethers). These compounds can pass biomembranes by free diffusion and thus exhibit a good bioavailability, when applied via skin, mucosal surfaces, inhalation, and ingestion [1]. Since the lipophilic compounds can disturb membrane permeability, a loss of ions, a reduction of the membrane potential, a collapse of the proton pumps, and depletion of the ATP pool can result when essential oils are applied in high concentrations [2]. Many factors influence the composition of essential oils. Environmental factors in particular (soil properties, water supply, sunlight, temperature) have a substantial effect on the quality and quantity of oil composition. Essential oils have gained special attention as raw materials for the production of perfumes, cosmetics, pharmaceuticals, and pesticides. In addition they are exploited in aromatherapy and in phytotherapy, and furthermore as spices and for nutrition [3]. In the last few decades, the biological properties of essential oils have been widely investigated [4]. Most essential oils contain compounds possessing antimicrobial properties, which are active against viruses, bacteria, and fungi [5,6]. Additionally of interest for food and pharmaceutical research, are their antioxidant and anti-inflammatory activities [3].

The objective of this work was to screen antimicrobial, antioxidant and anti-inflammatory activities of essential oils of selected aromatic plants from Tajikistan, which is a mountainous country in Central Asia with a rich flora including a large numbers of herbs and aromatic plants. Climate, high altitude, mountainous soil, and minerals favor plant growth, synthesis and accumulation of biological active secondary metabolites.

Some plants are endemic for Central Asia, such as *Ferula clematidifolia*, *Ferula foetida*, *Galagania fragrantissima*, *Hypericum scabrum*, *Hyssopus seravschanicus*, *Origanum tyttanthum,* and *Ziziphora clinopodioides*. Central Asia is the main speciation and diversification center of the genus Artemisia [7]. *Artemisia absinthium*, *Artemisia rutifolia*, and *Artemisia scoparia* were also included in this study. Other species occur in other European and Asian countries, such as *Achillea filipendulina*, *Artemisia absinthium*, *Mentha longifolia*, *Tanacetum vulgare*, *Tanacetum parthenium*, and *Hypericum perforatum*. *Anethum graveolens*, *Galagania fragrantissima* and *Ocimum basilicum* are used as vegetables. We had characterized the isolated essential oils quantitatively and qualitatively by gas-liquid chromatography-mass spectrometry (GLC-MS) before [8]. In this communication, we examined their antimicrobial, antioxidant, and anti-inflammatory activities to identify interesting candidates for a use in phytotherapy.

## 2. Experimental Section

### 2.1. Plant Material

The aerial parts of aromatic plants were collected from the central-southern part of Tajikistan during flowering and fruiting season of 2013. Voucher specimens of the plant material were deposited at the Department of Biology, Institute of Pharmacy and Molecular Biotechnology, Heidelberg University under accession numbers (Table 1).

**Table 1:** Origin of samples and yield of essential oils.

Plants		IPMB Accession Number	Yield of Essential oil, (in %; w/w)
Species	Family		
*Anethum graveolens* L.	Apiaceae	P8577	0.7–0.8
*Ferula clematidifolia* K.-Pol.	Apiaceae	P8580	0.2–0.5
*Ferula foetida* (Regel.)	Apiaceae	-	0.1–0.6
*Galagania fragrantissima* Lipsky	Apiaceae	P8578	0.1–0.2
*Achillea filipendulina* Lam.	Asteraceae	P8582	0.5–0.6
*Artemisia absinthium* L.	Asteraceae	P8583	0.2–0.5
*Artemisia rutifolia* Stephan ex Spreng.	Asteraceae	P8584	0.3–0.5
*Artemisia scoparia* Waldst. & Kit.	Asteraceae	P8585	0.2–0.5
*Tanacetum vulgare* L.	Asteraceae	P8586	0.3
*Tanacetum parthenium* (L.) Schultz-Bip.	Asteraceae	P8587	0.3
*Hypericum perforatum* L.	Clusiaceae	P8592	0.4
*Hypericum scabrum* L.	Clusiaceae	P8593	0.1
*Hyssopus seravschanicus* Pazij	Lamiaceae	-	0.9–1.0
*Mentha longifolia* (L.) Huds.	Lamiaceae	P8595	0.6–0.8
*Origanum tyttanthum* Gontsch.	Lamiaceae	P8596	0.7–0.9
*Ocimum basilicum* Linn.	Lamiaceae	P8597	0.5
*Salvia sclarea* L.	Lamiaceae	P8598	0.3–0.4
*Ziziphora clinopodioides* Lam.	Lamiaceae	P8599	0.7–0.8

The essential oils were isolated from the dried aerial parts of plants by hydrodistillation using the Clevenger type apparatus for 3 h [9]. The accession number of plants and yields of essential oils are summarized in Table 1.

### 2.2. Antimicrobial Activity

The essential oils were suspended in Tween-80 and screened against a gram-positive and a gram-negative bacterium at concentrations between 9.8 µg/mL and 20 mg/mL. The final Tween concentration did not exceed 0.5%. The tested bacteria were *Staphylococcus aureus* MRSA NCTC 10442 and *E. coli* ATCC 25922, provided courtesy of the Department of Infectious Diseases, Medical Microbiology and Hygiene, Heidelberg University, Heidelberg, Germany. The organisms were cultured on Columbia Agar supplemented with 5% sheep blood and in Müller-Hinton broth. The minimum inhibitory concentration (MIC) was obtained by means of broth microdilution following the method of CLSI (2012) with incubation at 35 °C for 18 h. For the determination of the minimum bactericidal concentration (MBC), 3 µL of each well with concentrations at and above the MIC was streaked on agar plate and incubated for 24 h. The least concentration of the oil killing at least 99.9% of the initial inoculum was considered the MBC. Tween-80 growth and sterility controls were included in the tests and ampicillin served as positive control. The tests were conducted in duplicate per plate and performed three times.

### 2.3. Antioxidant Activity

The antioxidant activity of the essential oils was evaluated by 2,2-diphenyl-1-picrylhydrazyl (DPPH), 2,2′-azino-bis-(3-ethylbenzthiazoline-6-sulfonic acid) (ABTS), and ferric reducing antioxidant power (FRAP) assays. DPPH, ABTS and FRAP assays were analyzed as described earlier by us [10,11].

### 2.4. Anti-Inflammatory Activity

The anti-inflammatory activity of the essential oils was evaluated by inhibition of the soybean 5-lipoxygenase (5-LOX) enzyme, as described earlier [10].

## 3. Results

The antimicrobial, antioxidant and anti-inflammatory activities of essential oils from *Anethum graveolens* (AG), *Ferula clematidifolia* (FC), *Ferula foetida* (FF), *Galagania fragrantissima* (GF) (Apiaceae), *Achillea filipendulina* (AF), *Artemisia absinthium* (AA), *Artemisia rutifolia* (AR), *Artemisia scoparia* (AS), *Tanacetum vulgare* (TV), *Tanacetum parthenium* (TP) (Asteraceae), *Hypericum perforatum* (HP), *Hypericum scabrum* (HSc) (Clusiaceae), *Hyssopus seravschanicus* (HS), *Mentha longifolia* (ML), *Origanum tyttanthum* (OT), *Ocimum basilicum* (OB), *Salvia sclarea* (SS), and *Ziziphora clinopodioides* (ZC) (Lamiaceae) were evaluated by using photometric DPPH, ABTS, FRAP determinations, antimicrobial and 5-LOX assays. The corresponding results are documented in Table 2 and Table 3.

**Table 2:** Minimum inhibitory concentrations (MIC) and minimum bactericidal concentrations (MBC) of selected essential oils.

Species	*E. coli* ATCC 25922		MRSA NCTC 10442	
	MIC (mg/mL)	MBC (mg/mL)	MIC (mg/mL)	MBC (mg/mL)
*Achillea filipendulina*	5	10	5	5
*Artemisia absinthium*	>20	>20	>20	>20
*Artemisia rutifolia*	10	20	5	20
*Artemisia scoparia*	2.5	5	1.250	2.500
*Ferula clematidifolia*	>20	>20	>20	>20
*Ferula foetida*	>20	>20	>20	>20
*Galagania fragrantissima*	>20	>20	0.039	0.078
*Hypericum perforatum*	5	5	1.250	2.500
*Hypericum scabrum*	>20	>20	>20	>20
*Hyssopus seravschanicus*	10	10	5	10
*Mentha longifolia*	5	10	10	20
*Ocimum basilicum*	>20	>20	>20	>20
*Origanum tyttanthum*	0.313	0.313	0.625	1.250
*Salvia sclarea*	>20	>20	>20	>20
*Tanacetum vulgare*	>20	>20	20	20
*Ziziphora clinopodioides*	5	5	10	10
Positive control: Ampicillin	0.004	0.008	0.008	0.016

**Table 3:** Antioxidant and anti-inflammatory activity of essential oils from Tajikistan.

Species	DPPH, IC_50_, mg/mL	ABTS, IC_50_, mg/mL	FRAP, µM Fe (II)/mg Sample	5-LOX Inhibition, IC_50_, µg/mL
*Achillea filipendulina*	4.83	2.01	214.2	221.3
*Anethum graveolens*	4.98	4.12	47.9	33.47
*Artemisia absinthium*	**1.35**	**0.87**	**338.9**	**56.6**
*Artemisia rutifolia*	7.91	0.25	74.2	75.6
*Artemisia scoparia*	2.55	0.28	43.1	184.3
*Ferula clematidifolia*	15.7	0.45	124.5	-
*Ferula foetida*	17.82	7.98	345.9	-
*Galagania fragrantissima*	8.13	4.74	67.2	7.34
*Hypericum perforatum*	3.71	0.48	98.25	-
*Hypericum scabrum*	6.69	5.67	22.5	-
*Hyssopus seravschanicus*	4.90	1.39	53.8	100.7
*Mentha longifolia*	2.31	0.67	76.9	28.14
*Ocimum basilicum*	5.94	7.98	51.6	-
*Origanum tyttanthum*	**0.28**	**0.12**	**699.2**	**14.78**
*Salvia sclarea*	12.50	5.03	54.0	not active
*Tanacetum parthenium*	4.82	0.96	-	-
*Tanacetum vulgare*	7.69	2.56	70.5	-
*Ziziphora clinopodioides*	5.12	0.79	66.9	33.12
Ascorbic acid	0.007	0.0055	1899.5	-

DPPH: 2,2-diphenyl-1-picrylhydrazyl. ABTS: 2,2′-azino-bis-(3-ethylbenzthiazoline-6-sulfonic acid). FRAP: ferric reducing antioxidant power.

At the concentrations tested, most essential oils have shown no or weak antimicrobial activity against MRSA NCTC 10442 and *E. coli* ATCC 25922. The essential oil of *Origanum tyttanthum* inhibits MRSA and *E. coli* with a medium antibacterial activity (MIC and MBC ranging between 312.5 and 1250 µg/mL). The essential oil of *Galagania fragrantissima* was more potent against MRSA. The growth of the bacteria was completely inhibited at concentrations of 39 µg/mL, whereas 78 µg/mL was sufficient to kill the cells.

Several of the oils exhibited a substantial antioxidant activity (Table 3). The IC_50_ values of antioxidant activities according to DPPH assay ranged between 0.28 and 17.82 mg/mL (in descending order: OT > AA > ML > AS > HP > AF > TP > HSe > AG > ZC > OB > HSc > TV > GF > SS > FC > FF). The essential oil of *Origanum tyttanthum* exhibited the strongest effect with an IC_50_ of 0.28 mg/mL.

The IC_50_ values of antioxidant activities ranged between 0.12 and 7.98 mg/mL by ABTS assay (in descending order: OT > AR > AS > FC > HP > ML > AA > TP > HSe > AF > TV > AG > GF > SS > HSc > OB > FF) (Table 3). Results of the ABTS assay also show that the essential oil of *Origanum tyttanthum* exhibited the strongest effect with an IC_50_ of 0.12 mg/mL.

Ferric reducing antioxidant power for essential oils were ranged between equivalent to 22.5 and 699.2 µM Fe (II)/mg in descending order: OT > FF > AA > AF > FC > HP >ML > AR > TV > GF > ZC > SS > HSe > OB > AG > HSc (Table 3).

Some of essential oils inhibit soybean 5-LOX (Table 3) with IC_50_ values between 7.34 and 221.3 µg/mL. The essential oil of *Galagania fragrantissima* exhibited the strongest effect with an IC_50_ of 7.34 µg/mL followed by that of another endemic plant, *Origanum tyttanthum* (14.8 µg/mL).

## 4. Discussion

The percentage of oil yield of investigated plants ranged between 0.1% and 1%. *Hyssopus seravschanicus* had the higher yield of essential oil (0.9%–1%). In contrast, *Hypericum scabrum* was less productive in essential oil extraction yield (0.1%).

The antimicrobial activity of the essential oil of *Origanum tyttanthum* is related to the presence of phenolic components such as carvacrol (34.3%–59.2%) and thymol (10.8%–46.4%) as the major components [12]. Thymol and carvacrol exhibited the highest antibacterial activity; they are known for their membrane-disturbing activities, as well as cell lysis [13]. Thymol and carvacrol interfere with the activity of cell wall enzymes like chitin synthase/chitinase as well as α- and β- glucanases [13].

The main constituent of the essential oil of *Galagania fragrantissima* was the aliphatic aldehyde, (2*E*)-dodecenal (83.6%) [14]. The advantage of (2*E*)-dodecenal is that it has hydrophobic alkyl (tail) chain and hydrophilic aldehyde group (head). Antibacterial activity of (2*E*)-dodecenal is correlated with physico-chemical damage to the cells, such as the disruption of the membrane and probably interference with proteins [15] and nucleic acids [6]. The 2*E*-dodecenal inhibited *Salmonella choleraesuis* (gram-negative bacterium) with an IC_50_ of 6.25 µg/mL [15].

The antioxidant potential of an essential oil depends on its composition. Phenolics and secondary metabolites with conjugated double bonds usually show substantial antioxidative properties [16]. In previous studies, we have already analyzed the chemical composition of the essential oils by GLC-MS in detail [8]. Most of the essential oils are dominated by oxygenated monoterpenes: alcohols (*Achillea filipendulina*), aldehydes (*Galagania fragrantissima*), ketones (*Anethum graveolens, Artemisia rutifolia, Hyssopus seravschanicus, Mentha longifolia, Ziziphora clinopodioides*), and esters (*Salvia sclarea*)*. Artemisia absinthium* and *Artemisia scoparia* predominantly contain monoterpene hydrocarbons. Phenolic terpenoids, such as thymol or carvacrol, are present in *Origanum tyttanthum* and *Mentha longifolia,* which would explain, that both plants exhibited the strongest antioxidant activity. These results are consistent with those previously reported by other authors [17,18], namely that the phenolic monoterpenes thymol and carvacrol which are predominant in *Origanum tyttanthum* are also responsible for the antioxidant activity of several other essential oils (e.g., *Mentha longifolia, Thymus serpyllus*) which contain them.

The enzyme 5-LOX is involved in the transformation of arachidonic acid into leukotrienes. Overproduction of leukotrienes causes inflammation. *Galagania fragrantissima* oil is dominated by unsaturated aldehyde ((2*E*)-dodecenal), which is responsible for anti-inflammatory activity. It is very electrophilic and can react with a variety of nucleophiles, such as amino groups either from proteins. Additionally, due to the structural similarities to fatty acids, aliphatic aldehydes (*trans*-2-decenal, dodecanal and decanal) have strong 5-lipoxygenase inhibitory activity [19,20].

The biological activities of the essential oil from *Origanum tyttanthum* may be attributed to the presence of phenols (carvacrol and thymol) as the major oil components [21]. The anti-inflammatory effects of oregano (carvacrol as major component) oil in mice with TNBS-induced colitis showed that some combinations lowered the amount of IL-1β and IL-6 cytokines [22]. In addition, it has been suggested that thymol has a potential as a non-steroidal anti-inflammatory drug [23].

## 5. Conclusions

Antimicrobial, antioxidant, and anti-inflammatory activities of the essential oils of 18 plant species from Central Asia were investigated *in vitro*. Essential oils of *Origanum tyttanthum* and *Galagania fragrantissima* exhibit substantial antimicrobial, antioxidant and anti-inflammatory activities. They are interesting candidates in phytotherapy.
